# Características da vizinhança em Belo Horizonte, Minas Gerais, Brasil: uma análise intraurbana usando o método de observação social sistemática

**DOI:** 10.1590/0102-311XPT206023

**Published:** 2025-01-13

**Authors:** Amanda Silva Magalhães, Amanda Cristina de Souza Andrade, Bruno de Souza Moreira, Solimar Carnavalli Rocha, Débora Moraes Coelho, Adalberto Aparecido dos Santos Lopes, Aline Dayrell Ferreira Sales, Amélia Augusta de Lima Friche, Waleska Teixeira Caiaffa

**Affiliations:** 1 Observatório de Saúde Urbana de Belo Horizonte, Universidade Federal de Minas Gerais, Belo Horizonte, Brasil.; 2 Instituto de Saúde Coletiva, Universidade Federal de Mato Grosso, Cuiabá, Brasil.; 3 Núcleo de Estudos em Saúde Pública e Envelhecimento, Universidade Federal de Minas Gerais/Fundação Oswaldo Cruz, Belo Horizonte, Brasil.; 4 Grupo de Estudos e Pesquisa em Ambiente Urbano & Saúde, Universidade Federal de Santa Catarina, Florianópolis, Brasil.; 5 Departamento de Medicina Preventiva e Social, Universidade Federal de Minas Gerais, Belo Horizonte, Brasil.; 6 Departamento de Fonoaudiologia, Universidade Federal de Minas Gerais, Belo Horizonte, Brasil.

**Keywords:** Características de Residência, Características da Vizinhança, Áreas de Pobreza, Saúde da População Urbana, Planejamento de Cidades, Residence Characteristics, Neighborhood Characteristics, Poverty Areas, Urban Health, City Planning, Características de la Residencia, Características del Vecindario, Áreas de Pobreza, Salud Urbana, Planificación de Ciudades

## Abstract

A observação social sistemática (OSS) é um método objetivo de mensuração das características físicas e sociais da vizinhança. O objetivo foi construir indicadores intraurbanos a partir do método de OSS e compará-los entre duas favelas e seus entornos em uma capital brasileira. Os indicadores simples foram calculados pelo método de estimadores de razão e agrupados em domínios. A análise de componentes principais gerou os indicadores compostos, sendo o número de componentes definido com base nas porcentagens da variância total explicada, e subdomínios criados quando dois componentes representavam o domínio. A consistência interna foi verificada pelo alfa de Cronbach, e os indicadores compostos transformados em escalas de 0 a 5. As comparações entre favelas e entornos foram realizadas pelo teste U de Mann-Whitney, considerando um nível de 5% de significância. Foram avaliados 373 segmentos de ruas em 63 vizinhanças. Para os domínios ruas, calçadas, sinalização e segurança foram observadas medianas maiores nos entornos, em comparação com as favelas, enquanto para os domínios interação social e problemas na vizinhança a mediana foi maior nas favelas. Os indicadores compostos têm potencial para identificar disparidades intraurbanas dentro da cidade e contribuir para a implementação de transformações urbanas visando aprimorar as condições de vida e saúde dos moradores.

## Abstract (en)

Systematic social observation (SSO) is an objective method of measuring the neighborhood physical and social characteristics. This study aimed to build intraurban indicators using the SSO method and compare them between two slums and their surroundings in a Brazilian capital. The simple indicators were calculated using the ratio estimator method, and grouped into domains. The principal component analysis generated the composite indicators, with the number of components defined based on the percentages of the total variance explained, and subdomains created when two components represented the domain. Internal consistency was verified by Cronbach’s alpha, and composite indicators were transformed into scales from 0 to 5. Comparisons between slums and surroundings were performed using the Mann-Whitney U test, considering a 5% significance level. We evaluated 373 street segments in 63 neighborhoods. For the streets, sidewalks, signage, and safety domains, higher medians were observed in the surrounding areas than in the slums. While for the domains of social interaction and problems in the neighborhood, the median was higher in the slums. The composite indicators have the potential to identify intraurban disparities within the city, and contribute to the implementation of urban transformations aimed at improving the residents’ living and health conditions.

## Abstract (es)

La observación social sistemática (OSS) es un método objetivo para medir las características físicas y sociales del vecindario. El objetivo fue construir indicadores intraurbanos con base en el método OSS y compararlos entre dos asentamientos infomales (*favelas*) y sus entornos en una capital brasileña. Los indicadores simples se calcularon usando el método de estimadores de razón, y se agruparon en dominios. El análisis de componentes principales generó los indicadores compuestos, con el número de componentes definidos en función de los porcentajes de varianza total explicada, y subdominios creados cuando dos componentes representaban el dominio. La consistencia interna se verificó mediante el alfa de Cronbach, y los indicadores compuestos se transformaron en escalas de 0 a 5. Las comparaciones entre asentamientos informales y entornos se realizaron mediante la prueba U de Mann-Whitney, considerando un nivel de significancia del 5%. Se evaluaron 373 tramos de calles en 63 vecindarios. Para los dominios calles, aceras, señalización y seguridad, se observaron medianas más altas en los entornos en comparación con los asentamientos informales. Mientras que, para los dominios de interacción social y problemas en el vecindario, la mediana fue mayor en los asentamientos informales. Los indicadores compuestos tienen el potencial de identificar disparidades intraurbanas dentro de la ciudad y contribuir a la implementación de transformaciones urbanas destinadas a mejorar las condiciones de vida y salud de los residentes.

## Introdução

A mensuração das características do ambiente urbano, em especial no nível da vizinhança, tem sido fomentada pela necessidade de informações para além das individuais, pois estas são insuficientes na compreensão dos determinantes da saúde ^1^. Além disso, as características da vizinhança podem contribuir para o entendimento das desigualdades em saúde, visto que o local de moradia é fortemente influenciado por posição social e etnia/raça ^1^ ^,^ ^2^.

As métricas na saúde urbana (informações, métodos e análises) são importantes para elucidar a relação da vizinhança com a saúde de seus moradores ^3^. Os métodos utilizados para obter essas informações são heterogêneos e suas particularidades devem ser consideradas, além da sua forma de mensuração, que pode ser realizada de maneira subjetiva ou objetiva ^4^. Entre os métodos subjetivos, destacam-se as entrevistas realizadas diretamente com os moradores e o uso de dados secundários, como do censo demográfico ^5^. Adicionalmente, no censo demográfico, também há a avaliação das características urbanísticas do entorno dos domicílios, o que amplia a gama de informações sobre o território ^6^.

Para mensuração objetiva mais proximal, o método de observação social sistemática (OSS) permite registrar, de forma válida e confiável, as características da vizinhança que não são capturadas pela percepção dos indivíduos, por informações censitárias e por outros macroindicadores. É um método reprodutível que permite obter dados quantitativos e qualitativos, e que pode ser aplicado de maneiras distintas ^7^ ^,^ ^8^, como em auditorias presenciais ou virtuais, por meio do Google Street View (https://www.google.com/maps/), por exemplo ^9^. No entanto, as auditorias presenciais, embora apresentando escalabilidade restrita, continuam sendo a forma de coleta de dados mais adequada para obter informações precisas em áreas urbanas de difícil acesso, como as favelas. Especialmente nessas áreas há dificuldade de obtenção de informações devido à irregularidade da ocupação e da malha viária, assim como da dificuldade de acesso aos locais mais vulneráveis. Uma vez obtidas as informações, outro desafio é utilizá-las de forma conjunta para construir escalas que possam categorizar os locais auditados ^7^ ^,^ ^8^ ^,^ ^9^.

As favelas são formas de ocupação irregular de terrenos de propriedade alheia para fins de habitação em áreas urbanas. De acordo com o Instituto Brasileiro de Geografia e Estatística (IBGE), as favelas expressam a desigualdade socioespacial da urbanização brasileira. Elas retratam a incompletude - no limite, a precariedade - das políticas governamentais e investimentos privados de dotação de infraestrutura urbana, serviços públicos, equipamentos coletivos e proteção ambiental aos sítios onde se localizam, reproduzindo condições de vulnerabilidade ^10^.

Estimativas recentes apontam que mais de 1 bilhão de indivíduos residem em favelas em todo o mundo; espera-se que este contingente populacional ultrapasse 2 bilhões até 2030 ^11^ ^,^ ^12^. Esse crescimento notável tem suscitado interesse e preocupação em âmbito internacional por parte de diversos atores, como governos, organizações e acadêmicos, levando os Objetivos de Desenvolvimento Sustentável das Nações Unidas a estabelecer metas para tornar as cidades e os assentamentos humanos inclusivos, seguros, resilientes e sustentáveis ^13^. Entretanto, a indisponibilidade de dados intraurbanos, principalmente em áreas de favela, prejudica os esforços políticos e o monitoramento dessas metas ^14^, além de manter disparidades existentes desconhecidas ou generalizadas ^12^ ^,^ ^15^.

Nesse contexto, é importante a construção de indicadores para a compreensão empírica dessas vizinhanças e para orientar políticas públicas ^7^ ^,^ ^12^ ^,^ ^15^. Portanto, o presente estudo teve como objetivos construir indicadores intraurbanos a partir do método de OSS e compará-los entre duas favelas e seus entornos em uma capital brasileira.

## Métodos

### Contexto

A presente pesquisa faz parte de um estudo epidemiológico maior com delineamento quase-experimental, multifásico e multimétodo, denominado *A Saúde dos Moradores em Zonas e Áreas Especiais de Interesse Social* (Projeto BH-Viva). O projeto foi iniciado em 2012 pelo Observatório de Saúde Urbana de Belo Horizonte da Faculdade de Medicina da Universidade Federal de Minas Gerais (UFMG) e se propõe a monitorar os efeitos das intervenções de requalificação urbana multifacetadas sobre o estado de saúde dos moradores de vilas e favelas de Belo Horizonte, Minas Gerais, Brasil. Mais detalhes sobre o Projeto BH-Viva podem ser obtidos em publicação prévia ^16^.

O Município de Belo Horizonte, cenário do estudo, é uma metrópole cuja lógica de produção e organização do espaço é similar à de megacidades. Apesar das políticas públicas sociais e urbanas voltadas, sobretudo, para territórios vulneráveis e implementadas nos últimos 30 anos ^17^, a capital mineira ainda apresenta uma parcela relevante da população, cerca de 480 mil habitantes (20%), vivendo em favelas ^18^.

**Figura 1:**
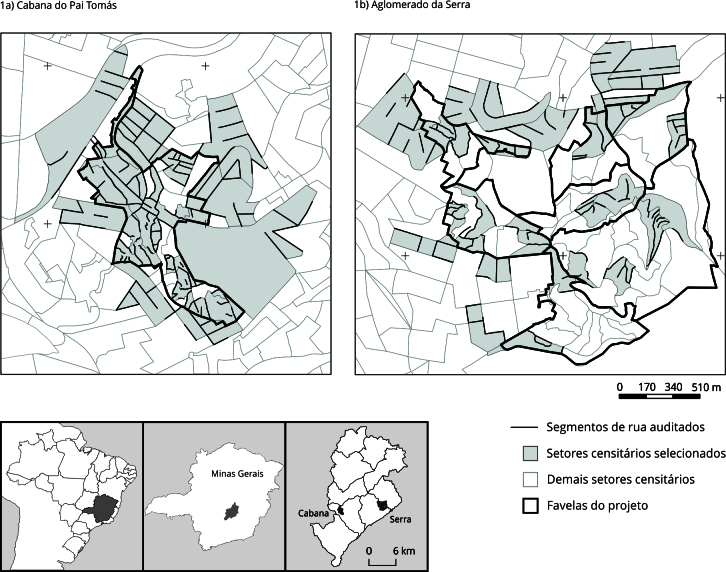
Mapa dos segmentos de ruas nos setores censitários das favelas e seus entornos amostrados na auditoria do Projeto BH-Viva. Belo Horizonte, Minas Gerais, Brasil, 2019.

A área de estudo incluiu o Aglomerado da Serra, aqui denominado AGS, e o Cabana do Pai Tomás (CPT), bem como os setores censitários contíguos a essas áreas, considerados como entornos das favelas (Figura 1). Em 2017 e 2018 foi realizado um inquérito domiciliar, com uma amostra de 1.194 domicílios distribuídos em 75 setores censitários da região (Tabela 1).

Fonte: elaborado por S. C. Rocha com base nos dados do Instituto Brasileiro de Geografia e Estatística ^11^ e da Prefeitura de Belo Horizonte ^18^.

**Tabela 1:** Características das favelas e seus entornos. Belo Horizonte, Minas Gerais, Brasil.

Características	Aglomerado da Serra		Cabana do Pai Tomás	
	Favelas	Entornos	Favelas	Entornos
Segmentos auditados	104	66	134	69
Setores censitários auditados	21	10	22	10
Setores censitários selecionados no inquérito domiciliar	29	11	22	13
Total de setores censitários (2010) *	64	27	34	23
Setores censitários por Índice de Vulnerabilidade da Saúde (%) *^,^**^,^***^,#^				
Baixo	0,0	72,0	0,0	19,0
Médio	1,6	20,0	8,8	62,0
Elevado	54,0	0,0	61,8	19,0
Muito elevado	44,4	8,0	29,4	0,0
Domicílios particulares permanentes (2010) *^,^***^,##^	11.365	5.585	5.815	3.379
População (2010) *^,^***^,###^	38.232	15.212	17.994	9.577
População por sexo (%) *^,^***^,###^				
Feminino	52,2	54,9	52,5	52,5
Masculino	47,8	45,1	47,5	47,5
População por faixa etária [anos] (%) *^,^***^,###,§^				
0-9	15,5	7,6	14,5	10,8
10-19	21,3	12,2	19,0	15,4
20-39	36,6	34,6	35,3	36,9
40-59	19,0	27,3	21,4	24,9
≥ 60	7,5	18,3	9,8	11,9
Total de setores censitários (2022) ^§§^	74	39	37	29
Domicílios particulares permanentes (2022) ***^,§§^	13.933	7.411	5.841	3.413
População (2022) ^***,§§^	36.488	15.591	14.704	7.838

Fonte: * Censo 2010 ^21^; ** Índice de Vulnerabilidade da Saúde 2012 ^20^; *** valores para o total de setores censitários por área; ^#^ dados faltantes em 1 setor censitário nas favelas e 3 nos entornos de Aglomerado da Serra, e 2 nos entornos do Cabana do Pai Tomás; ^##^ dados faltantes em 2 setores censitário nas favelas e 1 nos entornos do Aglomerado da Serra, e 2 nos entornos do Cabana do Pai Tomás; ^###^ dados faltantes devido à inexistência de construções residenciais para 1 setor censitário nas favelas e 1 nos entornos do Aglomerado da Serra; ^§^ dados faltantes para 7 indivíduos nos entornos do Aglomerado da Serra e 20 indivíduos nos entornos de Cabana do Pai Tomás; ^§§^ Censo 2022 ^19^.

O AGS é um conjunto de favelas localizado na regional Centro-Sul de Belo Horizonte, caracterizado por bairros com alto padrão de ocupação, residências verticalizadas e concentração de atividades econômicas, além de reunir e conciliar uma série de funções políticas, administrativas, sociais e culturais do município. A população estimada do AGS para o ano de 2022 foi de 36.488 moradores, distribuídos em 13.933 domicílios de 74 setores censitários, enquanto no seu entorno foi de 15.591 moradores, distribuídos em 7.411 domicílios de 39 setores censitários ^19^ (Tabela 1).

O CPT está localizado na regional Oeste, caracterizada pela presença de bairros mais antigos, mas que também se apresenta como uma área de expansão urbana. Em 2022, a população do CPT foi de 14.704 moradores, distribuídos em 5.841 domicílios de 37 setores censitários e, nos entornos, de 7.838 moradores distribuídos em 3.413 domicílios de 29 setores censitários ^19^ (Tabela 1).

Ambas as favelas possuem diferenciação em comparação a seus entornos. Conforme o Índice de Vulnerabilidade da Saúde de Belo Horizonte, essas áreas foram classificadas majoritariamente como de vulnerabilidade elevada e muita elevada. Esse índice é um indicador desagregado por setor censitário e composto por características socioeconômicas e de saneamento obtidas a partir das informações do censo demográfico de 2010 ^20^ ^,^ ^21^ (Tabela 1).

### Auditoria

A auditoria presencial pelo método de OSS foi realizada em outubro em 2019. Primeiramente, foram verificados os 75 setores censitários com entrevistas no inquérito domiciliar. Destes, foram selecionados os setores com três ou mais entrevistas, totalizando 63 setores (Tabela 1). Em seguida, foram identificadas todas as ruas com entrevistas do inquérito domiciliar, e para cada rua foi definido o segmento ^22^, totalizando uma amostra de 373 segmentos. Considerando as informações do inquérito domiciliar sobre características dos participantes e seu deslocamento em áreas próximas a sua residência, esses segmentos de rua foram agrupados em seus respectivos setores e definidos como vizinhanças ^7^.

O processo de espacialização, a definição dos segmentos de rua e a elaboração de *layout* final utilizado em campo foram realizados no software ArcGIS Desktop 10.5 (http://www.esri.com/software/arcgis/index.html).

### Instrumento

O instrumento utilizado na auditoria teve sua confiabilidade avaliada em estudo prévio, o qual se mostrou adequado para a observação de características com maior estabilidade temporal ^22^, e foi atualizado para o presente estudo por meio de uma revisão de literatura e das experiências acumuladas nas aproximações de campo. As 365 questões e subquestões foram alocadas em seis módulos: (1) físico; (2) caracterização dos imóveis; (3) estético; (4) serviços; (5) social e atividade física; (6) segurança. Além disso, para a coleta de dados foi elaborado um manual contendo informações detalhadas sobre os procedimentos de campo, com fotos exemplificando as questões e cada opção de resposta ^23^.

### Coleta de dados

Os observadores foram previamente treinados e realizaram estratégias de sensibilização para que os moradores das áreas investigadas tivessem conhecimento da pesquisa. Tanto na sensibilização quanto durante a auditoria, a equipe contou com o apoio de moradoras previamente selecionadas para que os acompanhassem durante a pesquisa.

Para as atividades de campo, cada observador tinha acesso ao manual e a um mapa contendo a localização e as especificações de início e término de cada segmento de rua. O instrumento foi dividido em duas partes, permitindo que o segmento fosse auditado por duplas, com cada parte do instrumento sendo preenchida simultaneamente por um observador e a comunicação entre eles permitida. O tempo médio de coleta foi de 27 minutos por dupla em cada segmento, variando de 9 a 100 minutos.

### Análise estatística

Os indicadores foram calculados conforme proposto por Costa et al. ^7^. Inicialmente, foi realizada uma análise exploratória de todos os itens obtidos na auditoria. A partir da prevalência e poder discriminatório das desigualdades espaciais, os itens elegíveis foram agrupados em oito domínios: ruas, calçadas, trânsito, estético, interação social, segurança, desordem física e serviços.

Os indicadores simples, representados pelas estimativas médias das razões observadas, foram calculados pelo método de estimadores de razão por meio de modelos de regressão linear, sendo obtidos também seus respectivos erros-padrão e considerado cada vizinhança como estrato. Para as variáveis categóricas, os indicadores foram construídos a partir da proporção média de segmentos de rua para cada item. No caso das variáveis contínuas, a partir da média de cada item foi utilizado o número de imóveis do segmento de rua como peso amostral.

Os indicadores compostos foram construídos com base em seus respectivos indicadores simples, sendo realizada a normalização e, quando necessário, a padronização para apresentar a mesma direção dentro do indicador. O método de análise de componentes principais gerou os indicadores compostos por meio da matriz de covariância, considerando valores acima de 0,30 para os coeficientes ^24^. O número de componentes foi definido com base nas porcentagens da variância total explicada, sendo criados subdomínios quando melhor representados por dois componentes. A consistência interna foi verificada pelo alfa de Cronbach, considerando correlações aceitáveis acima de 0,60 ^25^. Para facilitar a interpretação dos indicadores compostos estimados, eles foram transformados em uma escala de 0 a 5, de modo que um aumento na escala indica maior frequência dos itens.

Para descrever os indicadores simples e compostos foram calculadas as medianas e intervalos interquartílicos (IQ). Em seguida, foi realizada a comparação desses indicadores entre favelas e entornos por meio do teste não paramétrico de Mann-Whitney, considerando um nível de 5% de significância.

**Figura 2:**
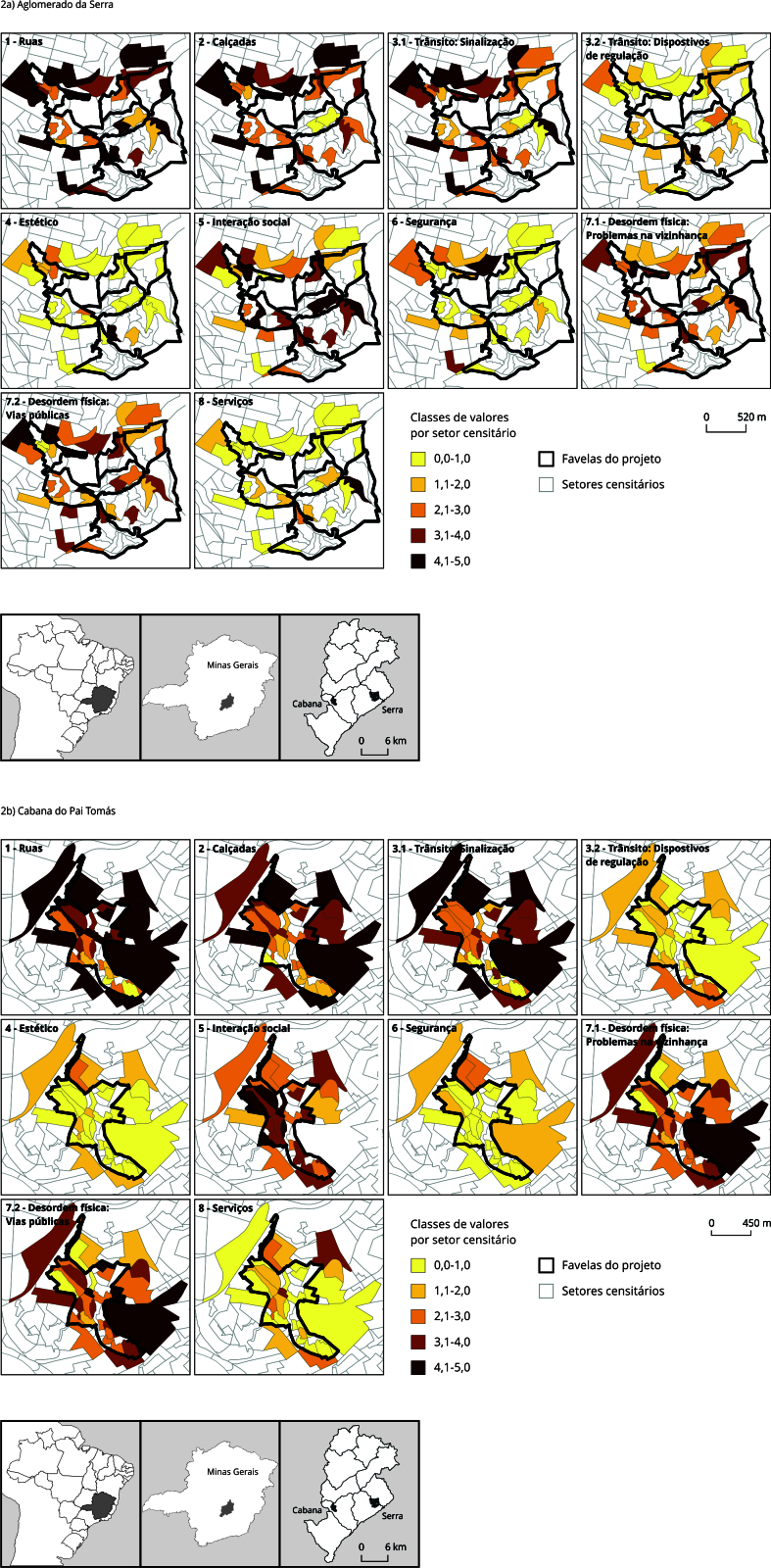
Distribuição espacial dos indicadores compostos das favelas e seus entornos amostrados na auditoria do Projeto BH-Viva. Belo Horizonte, Minas Gerais, Brasil, 2019.

Para a espacialização dos dados, foram utilizadas as bases cartográficas de setores censitários do *Censo Demográfico* de 2010, disponibilizadas pelo IBGE, e de favelas de Belo Horizonte, disponibilizadas pela Prefeitura de Belo Horizonte na plataforma digital BHmap (http://bhmap.pbh.gov.br/v2/mapa/idebhgeo#zoom=4&lat=7796893.0925&lon=609250.9075&baselayer=base). Todas as bases foram manipuladas utilizando-se o Sistema de Referência Geocêntrico para as Américas - SIRGAS 2000 (https://www.sirgas.org/pt/). Para cada indicador composto, os valores por setor censitário foram categorizados em cinco classes, sendo elas: 0,0-1,0; 1,1-2,0; 2,1-3,0; 3,1-4,0; 4,1-5,0, em uma escala de cores gradientes, gerando-se um total de 20 mapas temáticos, sendo 10 para cada uma das duas áreas de estudo (Figura 2).

As análises estatísticas foram realizadas por meio do software Stata 17.0 (https://www.stata.com) e os dados geográficos foram processados usando software ArcGIS Desktop 10.5 (http://www.esri.com/software/arcgis/index.html).

Fonte: elaborado por S. C. Rocha com base nos dados do Instituto Brasileiro de Geografia e Estatística ^11^.

### Aspectos éticos

O projeto foi aprovado pelos Comitês de Ética em Pesquisa da UFMG e da Secretaria Municipal da Saúde de Belo Horizonte (processo nº CAAE 11548913.3.0000.5149).

## Resultados

Foram avaliados 373 segmentos de rua com comprimento médio de 104 metros (± 44,8 metros), variando entre 22 e 245 metros, contidos em 63 vizinhanças. Cada vizinhança possuía em média 5,9 segmentos de ruas (± 1,4 segmentos). Quanto à distribuição entre as áreas de moradia, foram auditados 238 segmentos de ruas em 43 vizinhanças nas favelas e 135 segmentos de ruas em 20 vizinhanças nos entornos (Tabela 1).

Após análise exploratória, 63 itens foram elegíveis e agrupados em oito domínios: ruas (10 itens), calçadas (10 itens), trânsito (5 itens), estético (6 itens), interação social (5 itens), segurança (6 itens), desordem física (15 itens) e serviços (6 itens) (Quadro 1).

**Quadro 1:** Descrição dos itens dos indicadores da vizinhança por domínio.

INDICADORES POR DOMÍNIO	CATEGORIZAÇÃO/DEFINIÇÃO
1. Ruas	
Tipo de segmento	0: beco
1: via
Tipo de pavimentação	0: pé de moleque, piso cimentado, sem qualquer pavimentação ou em construção/reforma
1: asfalto
Condição de pavimentação *	Quantidade de mato, buraco e/ou saliência
Entrada e saída para veículos	0: não
1: sim
Interseção	0: rua/beco sem saída ou segmento único
1: três segmentos ou mais
Inclinação	0: moderado ou íngreme
1: plano
Meio-fio	0: não
1: sim
Sarjeta	0: não
1: sim
Galeria subterrânea	0: não
1: sim
Boca de lobo	0: não
1: sim
2. Calçadas	
Passeio	0: não
1: sim
Tipo de pavimentação	0: tijolo/bloco, terra/grama, cascalho/pedra britada ou outros
1: asfalto ou piso cimentado/concreto
Condição de pavimentação *	Quantidade de mato, buraco, saliência e/ou rachadura
Calçada em todo o segmento	0: não
1: sim
Calçada obstruída	0: não
1: sim
Rampa para carros	0: não
1: sim
Piso escorregadio	0: não
1: sim
Dispositivo de trânsito de pedestre *	Quantidade de rampa de acesso para deficientes, piso tátil direcional, piso tátil de alerta, corrimão com guarda-corpo, corrimão sem guarda-corpo e/ou escadaria
Meio-fio com menos de 20cm de altura	0: não
1: sim
Passeio com menos de 120cm de largura	0: não
1: sim
3. Trânsito	
Placa de trânsito	0: não
1: sim
Sinalização de estacionamento	0: não
1: sim
Faixa de tráfego	0: uma faixa
1: duas faixas ou mais
Sentido de circulação de veículos	0: sentido único
1: sentido duplo
Dispositivo de controle de tráfego *	Quantidade de canteiro central, grade de proteção, faixa de pedestre, lombada, passarela para pedestre, sinalização horizontal, sinalização de redução de velocidade, rotatória, sinal de parada obrigatória, radar eletrônico, semáforo e/ou sinal luminoso para pedestre
4. Estético	
Árvore, arbusto e/ou muda *	Quantidade de árvore, arbusto e/ou muda
Espaço aberto	0: nenhum
1: poucos ou muitos
Grafite *	Quantidade de grafite ou mural de pintura
Mensagem ideológica ou política *	Quantidade de mensagem ideológica ou política
Divulgação de trabalho informal *	Quantidade de divulgação de trabalho informal
Anúncios de comércio *	Quantidade de propaganda de comércio em geral
5. Interação social	
Pessoas sentadas, conversando ou interagindo	0: nenhuma
1: poucas ou muitas
Crianças [10 anos ou menos]	0: nenhuma 1: poucas ou muitas
Adolescentes [11-19 anos]	0: nenhum
1: poucos ou muitos
Adultos [20-59 anos]	0: nenhum
1: poucos ou muitos
Idosos [60 anos ou mais]	0: nenhum
1: poucos ou muitos
6. Segurança	
Poste de iluminação pública *	Quantidade de poste de iluminação pública
Muro com cacos de vidro e/ou pontiagudo *	Quantidade de muro com cacos de vidro e/ou portão ou muro pontiagudo/lança
Imóvel com cerca elétrica e/ou arame *	Quantidade de imóvel com cerca elétrica e/ou cerca de arame farpado/espiral
Imóvel com janela com grade *	Quantidade de imóvel com janela com grade
Aviso de alarme e/ou cão bravo *	Quantidade de aviso de propriedade protegida/alarme e/ou cão bravo
Câmera de segurança *	Quantidade de câmera de segurança ou aviso
7. Desordem física	
Lixo armazenado inadequadamente	0: não
1: sim
Lixo *	Quantidade de agulha, cigarro, lata, entulho, preservativo e/ou folhas
Cachorro desacompanhado	0: nenhum
1: poucos ou muitos
Dejeto de animal	0: nenhum
1: poucos ou muitos
Veículo abandonado	0: nenhum
1: poucos ou muitos
Objetos nos fios dos postes	0: nenhum
1: poucos ou muitos
Local com acúmulo de água	0: não
1: sim
Local degradado *	Quantidade de sinal de deterioração
Imóvel deteriorado	0: nenhum
1: poucos ou muitos
Imóvel em construção *	Quantidade de imóvel em construção
Lote malcuidado *	Quantidade de lote malcuidado
Pichação *	Quantidade de pichação
Ruído *	Quantidade de ruídos (tráfego veicular, publicidade móvel ou fixa e/ou construção civil)
Música	0: nenhum
1: pouca ou muita
Odor desagradável	0: não
1: sim
8. Serviços	
Recipiente para lixo	0: não
1: sim
Alimentação *	Quantidade de serviços de alimentação (armazém, mercearia, supermercado, carrinho de comida, lanchonete, frigorífico, padaria, cafeteria, restaurante e/ou sacolão)
Bar *	Quantidade de bar
Comércio *	Quantidade de comércio (centro comercial, *lan house*, banca de jornal, livraria, papelaria, loja de conveniência, loja de vestuário e acessórios, mecânica e acessórios automotivos, farmácia, *pet shop* e/ou depósito de material de construção)
Salão de beleza e/ou barbearia *	Quantidade de salão de beleza e/ou barbearia
Igreja ou centro religioso *	Quantidade de igreja ou centro religioso

Nota: para as variáveis categóricas, a categoria de interesse é representada pela categoria 1.

* Variáveis contínuas.

A Tabela 2 apresenta a comparação dos indicadores simples entre favelas e seus entornos. No domínio ruas, os entornos apresentaram valores medianos significativamente maiores em comparação com as favelas nos indicadores de tipo de segmento, tipo de pavimentação, entrada e saída para veículos, interseção e meio-fio.

**Tabela 2:** Comparação dos indicadores simples entre favelas e seus entornos. Belo Horizonte, Minas Gerais, Brasil, 2019.

Indicadores por domínio	Favelas		Entornos		Valor de p *
	Mediana	IQ	Mediana	IQ	
1. Ruas					
Tipo de segmento (via)	0,5	0,5	1,0	0,0	< 0,001
Tipo de pavimentação (asfalto)	0,7	0,6	1,0	0,1	< 0,001
Condição de pavimentação **	573,7	288,7	748,8	460,9	0,092
Entrada e saída para veículos (sim)	0,7	0,4	1,0	0,0	< 0,001
Interseção (três ou mais)	0,6	0,6	1,0	0,2	< 0,001
Inclinação (plano)	0,2	0,4	0,3	0,2	0,649
Meio-fio (sim)	0,8	0,5	1,0	0,0	< 0,001
Sarjeta (sim)	0,3	0,5	0,3	0,4	0,682
Galeria subterrânea (sim)	0,6	0,4	0,7	0,5	0,118
Boca de lobo (sim)	0,3	0,6	0,4	0,4	0,215
2. Calçadas					
Passeio (sim)	0,6	0,4	1,0	0,0	< 0,001
Tipo de pavimentação (asfalto, cimento ou concreto)	0,8	0,5	1,0	0,0	0,001
Condição de pavimentação **	970,5	1140,5	1982,7	1221,8	< 0,001
Calçada em todo o segmento (sim)	0,4	0,3	1,0	0,0	< 0,001
Calçada obstruída (sim)	0,6	0,4	0,6	0,4	0,682
Rampa para carros (sim)	0,5	0,4	0,8	0,4	< 0,001
Piso escorregadio (sim)	0,2	0,4	0,2	0,4	0,588
Dispositivo de trânsito de pedestre **	26,7	93,4	121,5	173,8	< 0,001
Meio-fio com menos de 20cm de altura (sim)	0,5	0,4	0,5	0,2	0,666
Passeio com menos de 120cm de largura (sim)	1,0	0,3	0,3	0,6	< 0,001
3. Trânsito					
Placa de trânsito (sim)	0,2	0,4	0,8	0,3	< 0,001
Sinalização de estacionamento (sim)	0,6	0,6	0,8	0,4	0,020
Faixa de tráfego (duas ou mais)	0,3	0,5	1,0	0,0	< 0,001
Sentido de circulação de veículos (sentido duplo)	0,8	0,4	1,0	0,1	0,036
Dispositivo de controle de tráfego **	13,3	46,9	85,5	116,6	< 0,001
4. Estético					
Árvore, arbusto e/ou muda **	70,0	122,7	603,2	748,5	< 0,001
Espaço aberto (poucos ou muitos)	0,6	0,7	0,7	0,3	0,435
Grafite **	31,1	51,0	28,5	62,0	0,717
Mensagem ideológica ou política **	15,6	46,8	2,4	33,4	0,164
Divulgação de trabalho informal **	16,7	34,4	17,5	53,7	0,347
Anúncios de comércio **	32,7	50,6	64,8	111,6	0,283
5. Interação social					
Pessoas sentadas, conversando ou interagindo (poucas ou muitas)	0,7	0,4	0,4	0,6	< 0,001
Crianças [10 anos ou menos] (poucas ou muitas)	0,5	0,4	0,2	0,3	< 0,001
Adolescentes [11-19 anos] (poucos ou muitos)	0,7	0,3	0,4	0,3	< 0,001
Adultos [20-59 anos] (poucos ou muitos)	1,0	0,2	0,9	0,2	0,380
Idosos [60 anos ou mais] (poucos ou muitos)	0,6	0,4	0,5	0,5	0,134
6. Segurança					
Poste de iluminação pública **	251,1	134,7	341,9	171,0	0,001
Muro com cacos de vidro e/ou pontiagudo **	56,2	55,6	214,4	115,4	< 0,001
Imóvel com cerca elétrica e/ou arame **	37,0	97,8	403,8	546,2	< 0,001
Imóvel com janela com grade **	324,6	184,8	437,5	292,4	0,017
Aviso de alarme e/ou cão bravo **	0,0	25,6	155,8	384,6	< 0,001
Câmera de segurança **	0,0	11,7	102,8	200,0	< 0,001
7. Desordem física					
Lixo armazenado inadequadamente (sim)	0,8	0,4	0,7	0,4	0,420
Lixo **	178,2	126,9	189,0	125,9	0,408
Cachorro desacompanhado (poucos ou muitos)	0,3	0,4	0,0	0,3	0,006
Dejeto de animal (poucos ou muitos)	0,7	0,7	0,5	0,5	0,100
Veículo abandonado (poucos ou muitos)	0,2	0,2	0,1	0,2	0,057
Objetos nos fios dos postes (poucos ou muitos)	0,5	0,5	0,1	0,3	< 0,001
Local com acúmulo de água (sim)	1,0	0,3	0,8	0,4	0,024
Local degradado **	156,0	174,8	94,0	207,7	0,141
Imóvel deteriorado (poucos ou muitos)	0,7	0,5	0,5	0,5	0,009
Imóvel em construção **	25,6	83,3	13,3	32,0	0,094
Lote malcuidado **	0,0	48,6	9,2	33,8	0,380
Pichação **	266,4	227,5	287,0	167,0	0,836
Ruído **	87,8	69,1	138,0	101,2	0,002
Música (pouca ou muita)	0,4	0,3	0,0	0,1	< 0,001
Odor desagradável (sim)	0,3	0,6	0,0	0,2	0,001
8. Serviços					
Recipiente para lixo (sim)	0,0	0,4	0,8	0,2	< 0,001
Alimentação **	34,4	67,2	17,1	56,8	0,248
Bar **	22,2	27,4	9,4	20,7	0,021
Comércio **	13,1	66,7	48,7	68,6	0,170
Salão de beleza e/ou barbearia **	11,8	32,5	19,7	42,4	0,381
Igreja ou centro religioso **	13,9	19,7	7,2	42,5	0,300

IQ: intervalo interquartílico.

Nota: as informações fornecidas entre parênteses são as categorias de interesse das variáveis categóricas.

* Teste U de Mann-Whitney;

** Indicador por 1.000 residências.

No domínio calçadas, as medianas dos indicadores de passeio, tipo de pavimentação, condição de pavimentação, calçada em todo o segmento, rampa para carros e dispositivo de trânsito de pedestre foram maiores nos entornos. E, como esperado, a mediana da presença de passeio com menos de 120cm de largura foi significantemente maior nas favelas que nos entornos (1,0 *versus* 0,3) (Tabela 2).

No domínio trânsito, todos os cinco indicadores foram significativamente diferentes entre as áreas de moradia e apresentaram maiores medianas nos entornos. No domínio estético, apenas o indicador de árvore, arbusto e/ou muda foi significativo, sendo maior nos entornos. Em relação ao domínio interação social, as favelas apresentaram medianas significativamente superiores às de seus entornos nos indicadores de pessoas sentadas, conversando ou interagindo, e presença de crianças e adolescentes. Já no domínio segurança, todos os seis indicadores foram significativamente diferentes entre as áreas de moradia e exibiram maiores medianas nos entornos (Tabela 2).

No domínio desordem física, foram observadas medianas maiores nas favelas para os indicadores de cachorro desacompanhado, objetos nos fios dos postes, local com acúmulo de água, imóvel deteriorado, música e odor desagradável. Por outro lado, observou-se mediana significativamente superior no indicador de ruído nos entornos em comparação com as favelas (138,0 *versus* 87,8) (Tabela 2).

No domínio serviços, dos seis indicadores elegíveis, apenas dois foram significativamente diferentes entre as áreas de moradia, sendo que o valor mediano do indicador de recipiente para lixo foi maior nos entornos, enquanto o do indicador de bar foi maior nas favelas (Tabela 2).

A Tabela 3 apresenta a comparação dos indicadores compostos entre favelas e seus entornos. Para o domínio ruas, a análise de componentes principais resultou em apenas um componente constituído de seis itens (tipo de segmento, tipo de pavimentação, entrada e saída para veículos, interseção, meio-fio e galeria subterrânea), com variância total explicada de 64,1% e alfa de Cronbach de 0,883. O valor mediano deste indicador foi significativamente maior nos entornos em comparação com as favelas (4,6 *versus* 3,2).

**Tabela 3:** Comparação dos indicadores compostos entre favelas e seus entornos. Belo Horizonte, Minas Gerais, Brasil, 2019.

Indicadores	Explicação (%)	Alfa de Cronbach	Favelas			Entornos		Valor de p *
			Mediana	IQ	Mediana	IQ	
1. Ruas	64,1	0,883	3,2	1,7	4,6	0,4	< 0,001
Tipo de segmento (via)							
Tipo de pavimentação (asfalto)							
Entrada e saída para veículos (sim)							
Interseção (três ou mais)							
Meio-fio (sim)							
Galeria subterrânea (sim)							
2. Calçadas	61,3	0,836	2,6	1,1	4,1	0,5	< 0,001
Passeio (sim)							
Tipo de pavimentação (asfalto, cimento ou concreto)							
Condição de pavimentação **							
Calçada em todo o segmento (sim)							
Rampa para carros (sim)							
3. Trânsito	74,4	0,767					
3.1. Sinalização			2,3	1,5	4,2	0,8	< 0,001
Placa de trânsito (sim)							
Sinalização de estacionamento (sim)							
Faixa de tráfego (duas ou mais)							
3.2. Dispositivos de regulação			0,9	1,3	1,1	0,8	0,132
Sentido de circulação de veículos (sentido duplo)							
Dispositivo de controle de tráfego **							
4. Estético	57,4	0,625	0,6	0,5	0,7	0,8	0,275
Grafite **							
Divulgação de trabalho informal **							
Anúncios de comércio **							
5. Interação social	57,3	0,812	3,7	1,2	2,3	1,2	< 0,001
Pessoas sentadas, conversando ou interagindo (poucas ou muitas)							
Crianças [10 anos ou menos] (poucas ou muitas)							
Adolescentes [11-19 anos] (poucos ou muitos)							
Adultos [20-59 anos] (poucos ou muitos)							
Idosos [60 anos ou mais] (poucos ou muitos)							
6. Segurança	64,1	0,883	0,4	0,5	1,6	1,4	< 0,001
Poste de iluminação pública **							
Muro com cacos de vidro e/ou pontiagudo **							
Imóvel com cerca elétrica e/ou arame **							
Imóvel com janela com grade **							
Aviso de alarme e/ou cão bravo **							
Câmera de segurança **							
7. Desordem física	56,4	0,717					
7.1. Problemas na vizinhança			3,4	1,1	2,3	1,3	< 0,001
Objetos nos fios dos postes (poucos ou muitos)							
Local com acúmulo de água (sim)							
Imóvel deteriorado (poucos ou muitos)							
7.2. Vias públicas			2,7	1,2	2,9	1,4	0,647
Lixo armazenado inadequadamente (sim)							
Cachorro desacompanhado (poucos ou muitos)							
Veículo abandonado (sim)							
Local degradado **							
8. Serviços	56,5	0,800	0,6	0,8	0,5	1,0	0,516
Alimentação **							
Bar **							
Comércio **							
Salão de beleza e/ou barbearia **							
Igreja ou centro religioso **							

IQ: intervalo interquartílico.

Nota: as informações fornecidas entre parênteses são as categorias de interesse das variáveis categóricas.

* Teste U de Mann-Whitney;

** Indicador por 1.000 residências.

O domínio de calçadas também resultou um único componente constituído por cinco itens (passeio, tipo de pavimentação, condição de pavimentação, calçada em todo o segmento e rampa para carros) e apresentou variância total explicada de 61,3% e alfa de Cronbach de 0,836. A mediana desse indicador também foi significativamente maior nos entornos do que nas favelas (4,1 *versus* 2,6) (Tabela 3).

Para o domínio trânsito, a análise de componentes principais resultou em dois componentes, ou seja, gerou dois subdomínios. O subdomínio sinalização foi constituído pelos indicadores de placa de trânsito, sinalização de estacionamento e faixa de tráfego, enquanto o subdomínio dispositivos de regulação foi composto pelos indicadores de sentido de circulação de veículos e dispositivo de controle de tráfego. A variância total explicada pelos dois subdomínios conjuntamente foi de 74,4% e o alfa de Cronbach foi de 0,767. Houve diferença significativa entre as áreas de moradia somente no subdomínio sinalização, sendo observada também maior mediana nos entornos do que nas favelas (4,2 *versus* 2,3) (Tabela 3).

No domínio estético, permaneceram três itens: grafite, divulgação de trabalho informal e anúncios de comércio. A variância explicada foi de 57,4% e o alfa de Cronbach de 0,625. Não foi observada diferença significativa nos valores medianos desse indicador entre as áreas de moradia (p = 0,275) (Tabela 3).

Diferentemente dos domínios anteriores, interação social e segurança foram compostos por todos os itens previamente elegíveis. A interação social apresentou mediana significativamente maior nas favelas do que nos entornos (3,7 *versus* 2,3). Neste indicador, 57,3% da variância total foi explicada e o alfa de Cronbach foi de 0,812. O valor mediano do domínio segurança foi significativamente maior nos entornos em comparação com as favelas (1,6 *versus* 0,4). A variância explicada foi de 64,1% e o alfa de Cronbach de 0,883 (Tabela 3).

O domínio de desordem física foi constituído por dois subdomínios, sendo que a variância total explicada conjuntamente por eles foi a menor entre todos os domínios (56,4%) e o alfa de Cronbach foi de 0,717. Nesse domínio, o subdomínio problemas na vizinhança foi representado pelos itens de objetos nos fios dos postes, local com acúmulo de água e imóvel deteriorado. Já o subdomínio vias públicas foi constituído pelos itens lixo armazenado inadequadamente, cachorro desacompanhado, veículo abandonado e local degradado. Houve diferença significativa entre as áreas somente no subdomínio problemas na vizinhança, sendo evidenciada maior mediana nas favelas que nos entornos (3,4 *versus* 2,3) (Tabela 3).

No domínio serviços, permaneceram cinco itens: alimentação, bar, comércio, salão de beleza e/ou barbearia e igreja ou centro religioso. A variância explicada foi de 56,5% e o alfa de Cronbach de 0,800. Não foi observada diferença significativa nos valores medianos desse indicador entre as áreas de moradia (p = 0,516) (Tabela 3).

A Figura 2 mostra a distribuição espacial, por setor censitário, dos indicadores compostos das áreas de favela deste estudo e seus respectivos entornos. Valores mais elevados são observados nas escalas de ruas, calçadas e sinalização em ambos os entornos. Nas favelas, destacam-se valores mais altos na escala de interação social. Por outro lado, as escalas de dispositivos de regulação, estético, segurança e serviços exibiram valores mais baixos em ambas as áreas, enquanto as escalas de desordem física apresentaram valores mais altos. Percebe-se que há algumas diferenças nos valores das escalas dos indicadores compostos não apenas entre as favelas e seus entornos, mas também no interior das favelas.

## Discussão

Neste estudo foram construídos indicadores compostos com propriedades psicométricas aceitáveis, capazes de discriminar características da vizinhança entre diferentes áreas da cidade. Os atributos da vizinhança, avaliados pelos domínios ruas, calçadas, sinalização e segurança foram mais frequentes nos entornos, enquanto nas favelas, foram os atributos avaliados pelos domínios interação social e problemas na vizinhança. Além disso, não foi observada diferença entre os locais de moradia nos domínios estético, vias públicas e serviços.

Os indicadores construídos foram capazes de revelar um pior cenário urbanístico das áreas informais da cidade ao evidenciar que suas ruas e calçadas apresentaram piores condições em comparação com o entorno. Esses achados revelam que, embora muito consolidadas e com grandes avanços, como o reconhecimento dos direitos dos moradores e os investimentos públicos realizados ^26^, essas áreas ainda apresentam muitos problemas, como infraestrutura urbana precária, o que interfere negativamente na promoção de atividade física, acessibilidade, mobilidade e segurança. Além disso, as ruas e calçadas assumem uma importância adicional, pois são atributos essenciais dos sistemas de transporte e mobilidade urbana ^27^ ^,^ ^28^.

Neste estudo, a análise de componentes principais gerou dois subdomínios no domínio trânsito. Apenas o escore do subdomínio sinalização foi significativamente diferente entre as áreas de moradia. O maior número de sinalizações de trânsito observadas nos entornos tem relação com a melhor infraestrutura de ruas e calçadas, e pode refletir maior mobilidade urbana. Diferente do observado nas favelas, que, assim como as barreiras impostas pela configuração espacial, que restringem o acesso veicular a ruas estreitas e periféricas, a menor oferta de transporte público pode limitar o fluxo de transporte nessas áreas e o acesso a recursos sociais e de promoção de saúde, como emprego, educação, limpeza urbana, recreação, assistência médica, entre outros ^29^ ^,^ ^30^ ^,^ ^31^. Também foram observados maiores escores no domínio relacionado à segurança da vizinhança nos entornos. Vale destacar que os itens avaliados no domínio de segurança não demonstram que os entornos são locais mais seguros, e sim que possuem mais itens que contribuem para a proteção dos imóveis e da vizinhança.

Em oposição aos achados descritos anteriormente, as favelas apresentaram maiores escores nos domínios interação social e problemas na vizinhança. Apesar das dificuldades estruturais encontradas nessas áreas, as favelas são frequentemente marcadas por ações sociais e pela alta interação social, possivelmente decorrente da proximidade espacial dos domicílios, vivacidade dos espaços públicos e do sentimento de pertencimento à comunidade ^32^. De fato, em estudo realizado em 2008-2009 na mesma cidade, foi descrita uma relação direta entre os marcadores de coesão social e viver em áreas vulneráveis. Residentes de áreas de maior vulnerabilidade relataram reconhecer praticamente todas as pessoas que passavam em frente às suas casas quando comparados com aqueles que residiam em áreas de baixa vulnerabilidade ^33^. Além disso, as favelas possuem alta densidade habitacional, sendo muitas de suas residências superlotadas, com quatro em cada 10 domicílios tendo mais de três pessoas por quarto, o que também contribui para a presença de mais pessoas nessas áreas ^10^ ^,^ ^34^.

Quanto à desordem física, a diferença observada entre as áreas de moradia foi no subdomínio problemas na vizinhança, composto pelos itens de objetos nos fios dos postes, local com acúmulo de água e imóvel deteriorado. Em relação à infraestrutura elétrica, ligações ilícitas e eletrocussões são comumente observados em áreas informais da cidade ^8^, e é notório que o acúmulo de água pode contribuir para a maior incidência de doenças associadas a questões hídricas, tais como infecciosas e vetoriais ^35^. De fato, a infraestrutura dessas áreas compõe um desafio complexo e multifacetado que requer abordagens abrangentes, capazes de contemplar um desenvolvimento social e econômico adequado para a promoção da saúde populacional ^29^ ^,^ ^36^.

Este estudo tem algumas limitações que devem ser reconhecidas. Os dados são provenientes de apenas duas favelas e seus entornos, selecionadas por possuírem dados do inquérito domiciliar, e assim não podem ser consideradas representativas das demais áreas informais da cidade. Além disso, o desenho do estudo impossibilita a observação da variação temporal de certos itens, como música, lixo, locais com acúmulo de água, interações sociais, entre outros, sendo que uma medição mais confiável exigiria mais de uma observação, com dias e horários diferentes para o mesmo segmento de rua, uma das dificuldades operacionais do método.

Por outro lado, o presente estudo adiciona informações sobre as características do contexto da vizinhança ao escasso conhecimento existente sobre análises intraurbanas envolvendo áreas de vulnerabilidade social, como favelas. Tal conhecimento pode contribuir para investigações mais detalhadas que considerem também o delineamento longitudinal. Além disso, o instrumento utilizado nessa auditoria é amplo e contempla diferentes características da vizinhança. Sendo assim, ele tem um grande potencial de uso para avaliar disparidades intraurbanas em outros municípios brasileiros. Por fim, apesar das limitações e outras formas de uso da OSS, a auditoria presencial tem se mostrado a mais consistente opção para mensuração de características da vizinhança em áreas de difícil acesso, contribuindo na identificação e monitoramento dos atributos que requerem atenção de gestores e planejadores urbanos.

A partir dos resultados deste estudo, foi possível descrever as características físicas e sociais de vizinhanças localizadas em favelas e seus entornos no Município de Belo Horizonte e identificar as disparidades presentes entre essas áreas. Os indicadores construídos poderão ser utilizados em estudos posteriores para compreender melhor a relação entre vizinhança e eventos em saúde dessa população. Adicionalmente, a compreensão dessas características pode contribuir para a adoção de ações e políticas mais assertivas, direcionadas à realidade da área.
